# Eyes on CHARGE syndrome: Roles of CHD7 in ocular development

**DOI:** 10.3389/fcell.2022.994412

**Published:** 2022-09-08

**Authors:** Laura A. Krueger, Ann C. Morris

**Affiliations:** Department of Biology, University of Kentucky, Lexington, KY, United States

**Keywords:** retina, eye, photoreceptors, CHD7, CHARGE syndrome, zebrafish

## Abstract

The development of the vertebrate visual system involves complex morphogenetic interactions of cells derived from multiple embryonic lineages. Disruptions in this process are associated with structural birth defects such as microphthalmia, anophthalmia, and coloboma (collectively referred to as MAC), and inherited retinal degenerative diseases such as retinitis pigmentosa and allied dystrophies. MAC and retinal degeneration are also observed in systemic congenital malformation syndromes. One important example is CHARGE syndrome, a genetic disorder characterized by coloboma, heart defects, choanal atresia, growth retardation, genital abnormalities, and ear abnormalities. Mutations in the gene encoding Chromodomain helicase DNA binding protein 7 (CHD7) cause the majority of CHARGE syndrome cases. However, the pathogenetic mechanisms that connect loss of CHD7 to the ocular complications observed in CHARGE syndrome have not been identified. In this review, we provide a general overview of ocular development and congenital disorders affecting the eye. This is followed by a comprehensive description of CHARGE syndrome, including discussion of the spectrum of ocular defects that have been described in this disorder. In addition, we discuss the current knowledge of CHD7 function and focus on its contributions to the development of ocular structures. Finally, we discuss outstanding gaps in our knowledge of the role of CHD7 in eye formation, and propose avenues of investigation to further our understanding of how CHD7 activity regulates ocular and retinal development.

## 1 Introduction

Vision, one of our major senses, is considered the largest contributor to perception of the world around us (Pike, 2012). The visual system requires signals to be received by the eye, processed through the retina, and interpreted by the brain. When development of the visual system or eye structures are disrupted, congenital eye defects and pediatric visual impairment may result. Pediatric visual impairment can be life altering, affecting motor, language, emotional, social, and cognitive development (Wilkinson and Trantham, 2004; West, 2002; Berit Augestad, 2007; Sabra et al., 2009; Rainey et al., 2016). Disruptions in eye development and congenital eye defects are often found as part of larger syndromic disorders, including CHARGE syndrome, a genetic disorder characterized by coloboma, heart defects, choanal atresia, growth retardation, genital abnormalities, and ear abnormalities.

Development of the visual system is highly conserved among vertebrates (Stenkamp, 2015). A significant amount of work has been completed to better understand pathways involved in ocular morphogenesis and retinal development, however many questions remain about the role and mechanism of individual genes in these processes. In this review, we will provide an overview of the processes of vertebrate ocular morphogenesis, retinal development, and photoreceptor differentiation, and will compare these processes in zebrafish and mouse, two widely used animal models for development biology research, as well as humans to highlight similarities and differences across vertebrates. Next, we will describe some of the congenital disorders that result when these processes are disrupted, with a particular focus on CHARGE syndrome. We will discuss CHD7 function and its contributions to the development of ocular structures, and the potential roles of two of its downstream targets (Sox4 and Sox11) in visual system development. Finally, we will highlight outstanding gaps in our knowledge of CHD7 function, and avenues of investigation to further our understanding of how CHD7 activity regulates ocular and retinal development.

## 2 Vertebrate ocular development

Development of the vertebrate eye and ocular structures is a result of the organized interactions of neuroectoderm (forms the retina and retinal pigment epithelium), surface ectoderm (forms the lens), and periocular mesenchyme made up of mesoderm and cranial neural crest cells (form the ocular blood vessels and anterior ocular structures).

**FIGURE 1:**
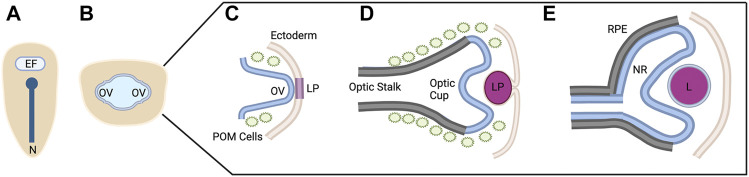
Vertebrate Ocular Development. Schematic representation of ocular development stages from eye field specification to bi-layered optic cup formation **(A–E)**. This process occurs between 12 and 24 hpf in zebrafish, E8-10 in mouse, and from ∼4 to 7 weeks in human. See text for process details. EF, eye field; N, notochord; OV; optic vesicle; LP, lens placode/pit; POM, periocular mesenchyme; RPE, retinal pigment epithelium; NR, neural retina; L, lens. Figure created with Biorender.com.

Briefly, early in vertebrate development, a single eye field is specified at the border of the anterior neural plate (Figure 1A) (Li et al., 1997). Cells within this eye field express known eye field transcription factors (EFTFs) including Pax6, Rax, Otx2, Six3, and Lhx2, among others; the eye field contains all necessary progenitor cells to form the neural portions of the eye (Zuber et al., 2003). Disruptions in the development of the eye field result in defects such as microphthalmia (small eye) or anophthalmia (single eye or no eyes) (Loosli et al., 2003; Voronina, 2003).

The eye field is subsequently separated into two domains through the action of Nodal/TGF-Β and Sonic hedgehog (Shh) signaling as the midline of the embryo is established (Echelard et al., 1993; Zhang et al., 1998). Disruptions in these signaling pathways and/or abnormal segregation of the eye field result in holoprosencephaly and cyclopia (Belloni et al., 1996; Roessler et al., 1996; Maity et al., 2005). The two separate eye fields evaginate away from the midline, forming the optic vesicles (Figure 1B) (Svoboda and O’Shea, 1987; Rembold et al., 2006). During this evagination process, the neural retina, retinal pigment epithelium (RPE), and optic stalk begin to be specified. The distal portion of the optic vesicle forms the neural retina, expressing the homeobox gene Vsx2; the dorsal proximal portion forms the RPE, expressing the transcription factor Mitf; and the ventral proximal portion forms the optic stalk, expressing Pax2 (Svoboda and O’Shea, 1987; Schmitt and Dowling, 1994; Burmeister et al., 1996; Nguyen and Arnheiter, 2000; Kagiyama et al., 2005).

Evagination of the optic vesicle also allows for the interaction with the surface ectoderm. This interaction leads to the thickening of the surface ectoderm which eventually forms the lens placode and is the initial step in lens formation (Figure 1C) (Hendrix and Zwaan, 1975; Huang et al., 2011). At the same time, the optic vesicle invaginates, forming the bilayered optic cup (Figure 1D). This invagination is a result of distinct cellular events in each of the layers. The distal layer shapes the presumptive retina through cellular basal constriction and retinal rim involution (Martinez-Morales et al., 2009; Kwan et al., 2012; Nicolás-Pérez et al., 2016; Sidhaye and Norden, 2017). The RPE is shaped by flattening of cells in the medial layer that surround the outside and rim of the retina (Figure 1E) (Li et al., 2000; Miesfeld et al., 2015; Cechmanek and McFarlane, 2017).

Furthermore, the optic vesicle and optic cup are receiving extrinsic signals from the periocular mesenchyme. It has been shown that periocular mesenchyme, consisting of mesoderm and neural crest cells, begins to migrate with the optic structures at the stage of evagination of the optic vesicle (Gage et al., 2005; Langenberg et al., 2008; Bryan et al., 2020). These periocular mesenchyme cells supply the signals for the specification of the RPE and differentiation of the optic stalk, in addition to migrating into the optic cup to become anterior ocular structures such as iris stroma and corneal endothelium (Gage et al., 2005).

As optic cup invagination is occurring, at the ventral portion near and within the optic stalk, an opening known as the optic fissure forms. Fissure formation is the result of the ventral edges of the optic cup and optic stalk approaching each other during invagination but remaining separated. Control of fissure formation is both intrinsic to cells of the optic cup in addition to contributions from surrounding tissue by Hedgehog, retinoic acid, and Bmp signaling, among others (Bernstein et al., 2018; Gordon et al., 2018). This opening allows space for the entrance of developing hyaloid vasculature and exit of retinal ganglion cell axons that bundle together to form the optic nerve. The fissure eventually closes to encapsulate these structures. The underlying molecular events that mediate fissure closure are a strong area of current research, however the general sequence of events is widely accepted. The closure process includes apposition of the edges of the fissure, followed by basement membrane rearrangement then epithelial remodeling and tissue fusion, and finally intercalation of tissue (Bernstein et al., 2018). Additionally, periocular mesenchyme cells are required for fissure closure. Specifically, a subset of neural crest cells and endothelial cells seem to be necessary for basement membrane rearrangement (James et al., 2016; Cao et al., 2018; Gestri et al., 2018; Weaver et al., 2020). When optic fissure formation or closure is disrupted, the birth defect known as coloboma results (Chang et al., 2006). Coloboma can occur solely or as part of larger syndromic disorders and involves different portions of the eye, resulting in visual impairment of varying severity depending on its location and size; coloboma will be discussed further in a later section.

Finally, the anterior structures of the eye,—cornea, iris and ciliary body—are forming during and after optic cup completion. The cornea forms with interactions between the surface ectoderm and migrating periocular mesenchyme while the iris and ciliary body are formed at the edges of the optic cup from proliferating periocular mesenchyme cells (Cvekl and Tamm, 2004; Davis-Silberman and Ashery-Padan, 2008).

## 3 Vertebrate retinal development

**FIGURE 2:**
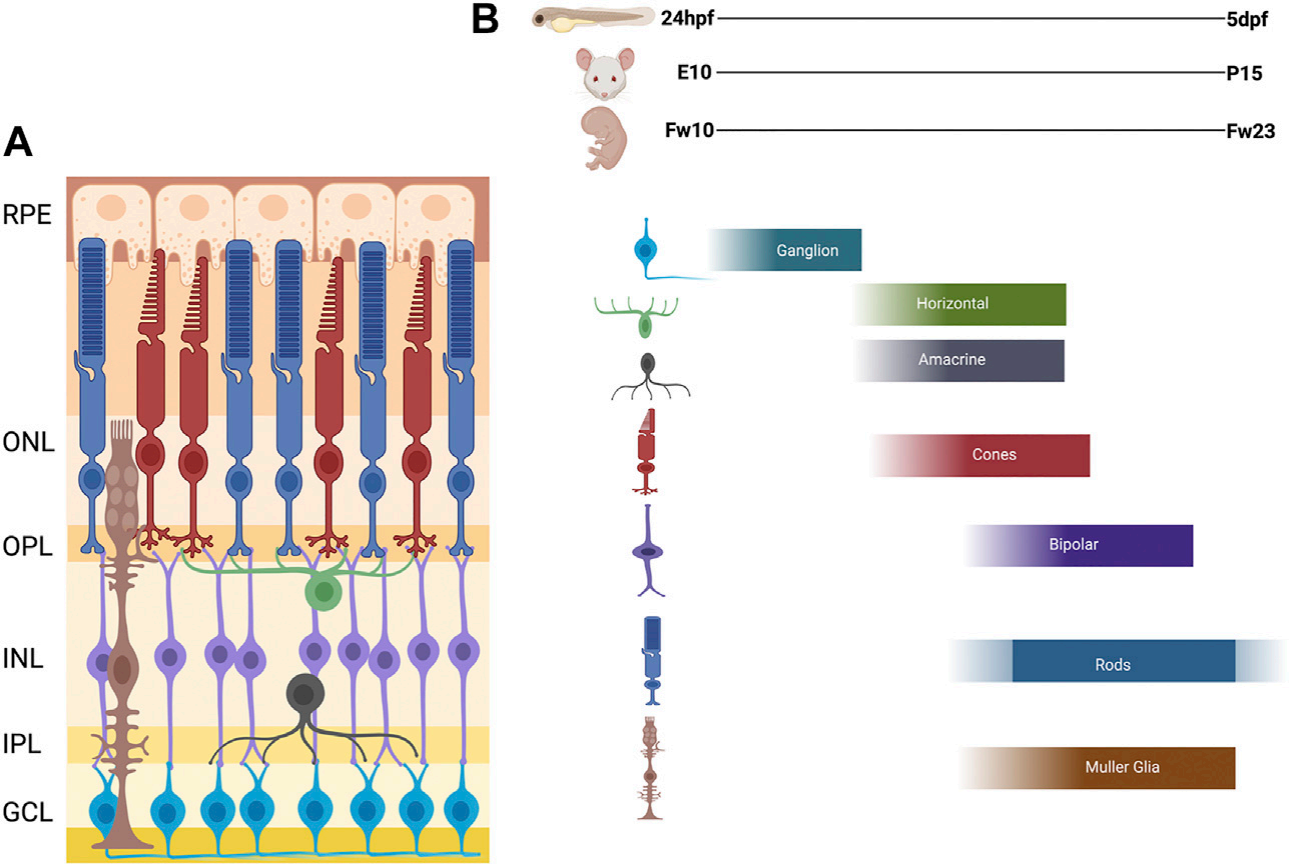
Schematic of Vertebrate Retina and Order of Retinal Cell Differentiation. **(A)** Diagram of vertebrate retina. The retina is composed of three nuclear layers: Outer Nuclear Layer (ONL), the Inner Nuclear Layer (INL), and the Ganglion Cell Layer (GCL), and two plexiform layers: Outer plexiform later (OPL) and Inner Plexiform Layer (IPL). The synapses for the photoreceptors, horizontal, bipolar are in the OPL and amacrine, bipolar, and ganglion are in the IPL. **(B)** The order of retinal cell differentiation is conserved across zebrafish, mouse and human. Most of this cell differentiation occurs between 24 h post fertilization (hpf) to 5 days post fertilization (dpf) in zebrafish, embryonic day 10 (E10) to post-natal day 15 (P15) in mouse, and fetal week 10 (Fw10) to fetal week 23 (Fw23) in humans. Figure created with Biorender.com.

As stated above, the inner layer of the of the optic cup forms the neural retina. The vertebrate neural retinal progenitor pool proliferates and differentiates into six neuronal cell types; retinal ganglion cells (RGCs), amacrine cells, horizontal cells, bipolar cells, and rod and cone photoreceptors; as well as one intrinsic glial cell type, the Müller glia. Within these broad cell categories, several subtypes have been described for some retinal cell types, the number of which can vary considerably among different vertebrate species. For example, at least 18 subtypes of RGCs have been described in the primate and human retina (Kim et al., 2021). Other cell types, such as astrocytes, microglia, and cells of the retinal vasculature, are generated outside the retina and subsequently migrate in. The retinal cells are organized in a conserved fashion across all vertebrates in three nuclear layers and two plexiform layers. The outer nuclear layer (ONL) contains the light sensitive rod and cone photoreceptors which then synapse with bipolar cells and horizontal cells in the outer plexiform layer (OPL). The inner nuclear layer (INL) contains the cell bodies of the bipolar, horizontal, amacrine, and Müller glial cells. The bipolar and amacrine cells synapse with retinal ganglion cells in the inner plexiform layer (IPL). The ganglion cell layer (GCL) contains the cell bodies of the retinal ganglion cells, whose axons then exit the retina and bundle together, forming the optic nerve, which transmits the visual signal to the brain. Müller glia span the entire retina and provide structural and functional support to the other retinal cells. This principal glial cell of the retina maintains architectural organization in addition to synthesizing and phagocytosing necessary products for retinal function and homeostasis (Figure 2A).

Decades of work in the retina have shown that all these retinal cell types differentiate from a single population of multipotent retinal progenitor cells (Turner and Cepko, 1987; Holt et al., 1988; Wetts and Fraser, 1988; Turner et al., 1990; Jensen and Raff, 1997). Furthermore, the spatiotemporal pattern of development and differentiation of the distinct retinal cells is largely conserved across vertebrates (Hu and Easter, 1999; Bassett and Wallace, 2012).

In zebrafish, formation of the bilayered optic cup is completed around 20 h post fertilization (hpf) and the inner neuroepithelial layer is the starting place for retinal cell differentiation (Schmitt and Dowling, 1994). Retinal neurogenesis begins in a small region of the ventronasal retinal neuroepithelium adjacent to the optic nerve (often referred to as the ventral patch). The ganglion cells are the first cell type to differentiate, exiting the cell cycle in a fan-shaped pattern that spreads from the ventral to dorsal retina (Schmitt and Dowling, 1999; Stenkamp, 2007). The cells of the INL follow the same fan-shaped pattern of differentiation between 36 hpf and 4 days post fertilization (dpf) for amacrine, horizontal, and bipolar cells (Schmitt and Dowling, 1999; Jusuf and Harris, 2009; Connaughton, 2011). Overlapping with this period, photoreceptors also begin to develop, with cone photoreceptors appearing first followed by rod photoceptors. Whereas cone photoreceptors differentiate in a pattern similar to the other retinal cell types (Suzuki et al., 2013), rod photoreceptor differentiation proceeds in an irregular pattern from the ventral patch to the dorsal and central retina (Raymond et al., 1995). In the post-embryonic retina, rods are primarily derived from a multipotent stem cell/Müller glia progenitor pool in the INL. These progenitors proliferate and migrate through the INL to ONL where they transition based on specific gene expression from progenitors to rod precursors, then to rod photoreceptors (Bernardos et al., 2007; Morris et al., 2008a, Morris et al., 2008b). The last cells to differentiate are the Müller glia (Turner and Cepko, 1987; Peterson et al., 2001).

The temporal progression of retinal neurogenesis is mostly conserved between mouse and zebrafish with specific timepoints of early born cells (ganglion cells, amacrine, horizontal and cone photoreceptors) developing between embryonic day 10 (E10) and postnatal day 1 (P1). The late born cells (rod photoreceptors, bipolar cells, and Müller glia) develop in the first 14 postnatal days (P0-P14) (Young, 1985; Marquardt and Gruss, 2002). In humans, this process occurs between fetal week 10–18 for the early born cells and fetal week 18–23 for the late born cells with some continued differentiation of photoreceptors occurring after birth (Figure 2B) (Aldiri et al., 2017; Hoshino et al., 2017).

While the cell type differentiation is complete in the mouse by 14 days after birth and in humans several months after birth, the zebrafish retina displays continual growth, which occurs from a persistently neurogenic area at the retinal periphery. This area, the ciliary marginal zone (CMZ), contains a pool of stem cells that allow for the continual addition of retinal cells as the zebrafish grows, enabling the retina to maintain appropriate cell density as the eye cup expands along with the body of the zebrafish (Stenkamp, 2007; Reinhardt et al., 2015). Furthermore, the zebrafish has the unique ability to respond to retinal damage with regeneration of retinal cells. In this case, Müller glia respond to damage by de-differentiating and asymmetrically dividing to produce multipotent stem cells that can regenerate all the lost retinal cell types (Yurco and Cameron, 2005; Sherpa et al., 2008, 2014).

## 4 Photoreceptor development

Vision requires that light be captured from the world around us. This process entails anterior structures of the eye (cornea, pupil, iris, and lens) to allow for light to enter, pass through, and focus correctly on the retina. Light hitting the retina must then be captured by the light sensitive cells, the photoreceptors. Photoreceptors can be broadly divided into two subtypes—the rods and cones. Rods are highly sensitive (able to detect a single photon of light) and mediate dim light vision (and peripheral vision in humans), whereas cones mediate daytime and color vision. Most mammals have two cone subtypes which are maximally sensitive to medium and long wavelenths of light. Humans possess three cone subtypes (short-, medium-, and long-wavelength sensitive cones). Zebrafish possess four cone subtypes (short-, medium-, long- and UV-sensitive cones). Photoreceptors have a distinct morphology to allow for this light sensitivity (Lamb et al., 2007; Molday and Moritz, 2015). Rod and cone photoreceptor cells have five main regions: the outer segment, connecting cilium, inner segment, nuclear body, and axonal and synaptic region. Outer segments contain membranous disks (rods) or folds (cones) which enclose opsins, the light-sensitive G-protein coupled receptor proteins responsible for the detection of light. The photopigment molecule of rods, rhodopsin, allows for the detection of dim light while the opsins of cones (red, green, and blue) allow for color vison and higher visual acuity. The outer segments of rods have a long cylindrical shape whereas cones have a shorter conical shape. Capturing of light in these photopigment molecules of the outer segment initiates the phototransduction pathway allowing for light to be converted to an electrical signal that is transmitted to the second-order neurons of the retina. The connecting cilium connects the outer and inner segments and provides a channel to allow for important proteins to pass from the cell body and nuclear region to and from the outer segment. The synaptic region is the site of transfer of neurotransmitters to retinal bipolar cells and the horizontal neurons.

The development of the outer segments of photoreceptors and their contained structures and proteins are essential for visual function. The outer segment resembles the structure of a primary sensory cilia and develops in a similar manner. First, during initial photoreceptor development, the axoneme/cilium projects from its basal body located in the inner segment. This primitive cilium contains vesicles and tubules of “morphogenetic material” that will establish other structures of the outer segments. Second, the apical region of the cilium enlarges as the material is used to form beginnings of disks. Third, these primitive disks are then remodeled and reoriented to their final transverse position. Recent work suggests that the nascent photoreceptor outer segment disks are similar in composition to ciliary ectosomes (small membranous vesicles that bud off from a cell’s primary cilium); these ecotomal structures form at the base of the photoreceptor cilium and are retained by structural proteins at the disk rim rather than being released into the extracellular space (Spencer et al., 2020). As a result of these steps the outer segment is formed by morphogenetic and differentiation events of the distal region of the primitive cilia. The undifferentiated basal portion remains continuous with the basal body and becomes the connecting cilium (de Robertis, 1956; Horst et al., 1990). Outer segment morphogenesis has been detected starting at 60–63 hpf in zebrafish and P8-9 in mice and continues until adult dimensions are met (de Robertis, 1956; Branchek and Bremiller, 1984; Obata and Usukura, 1992; Crespo and Knust, 2018). Outer segments are further maintained throughout life by undergoing constant renewal. New disks are continually being synthesized at the base of outer segments which leads to elongation of the outer segment. At the distal outer segment of the photoreceptor, the oldest disks are then shed and are phagocytosed by the RPE (Young, 1967).

Although photoreceptor function is conserved across species, the spatial organization and distribution of rods and cones varies based on evolutionary strategies. In humans, this means a peripheral retina that is rod dominant with a central region, the macula, which has a higher density of cones and a fovea with only cones allowing for our high visual acuity (Ahnelt, 1998). In contrast, nocturnal mice are rod dominant throughout their retina and only contain two types of cones, short and medium wavelength (Carter-Dawson and Lavail, 1979). The zebrafish has a cone-rich retina, possessing 60% cones, similar to the macula of humans and reflecting their diurnal lifestyle (Fadool, 2003; Allison et al., 2010; Zimmermann et al., 2018). In addition, zebrafish contain a fourth cone subtype that is sensitive to ultraviolet light. The four cones in zebrafish are arranged in a unique geometric mosaic pattern in which red/green double cones are always located next to blue cones on the red cone side and UV cones on the green cone side. Furthermore, in adult zebrafish, four rods form a square surrounding the UV cones (Fadool, 2003).

Much work has been done to investigate the many different signals and pathways involved in the process of photoreceptor development. This work has shown that progenitor cells are not limited to a specific path but instead during proliferation or post mitotically, precursors encounter different specification events leading to their fate. Furthermore, the pathway and sequence of events are generally conserved among vertebrates (Cepko et al., 1996; Wong and Rapaport, 2009). These events that control specification are from intrinsic gene expression programs as well as extrinsic signals and can be both positive (acquiring an event/signal) or negative (restricting another event/signal).

Briefly, the photoreceptor progenitor pool is fist specified by the expression of the transcription factors Otx2 and Crx*.* When Otx2 is absent, photoreceptors do not develop and instead there is an increase in amacrine like cells (Chen et al., 1997; Furukawa et al., 1997; Nishida et al., 2003) while defective photoreceptor development and degeneration occurs when Crx is absent (Furukawa et al., 1999). Additionally, NeuroD, a bHLH transcription factor, is present in the rod progenitor pool of the inner nuclear layer as well as cone progenitor pool and has been implicated in controlling cell cycle regulation and cellular proliferation of precursors (Ochocinska and Hitchcock, 2007; Nelson et al., 2008). This photoreceptor precursor pool is then further specified into rod and cone subtypes.

Expression of the transcription factor Nrl is required for the rod photoreceptor fate (Mears et al., 2001). Nrl, a basic leucine zipper transcription factor, promotes the expression of important downstream rod photoreceptor genes including the orphan nuclear receptor Nr2e3, which is responsible for activating other rod specific genes as well as repressing cone specific genes like S-opsin (Kumar et al., 1996; Cheng et al., 2011; Kautzmann et al., 2011). Defects in both Nrl and Nr2e3 lead to non-functional rods in mice and the genetic disorder of S-cone syndrome is attributed to loss of Nr2e3 in humans and results in a fate switch from rods to S-cones (Haider et al., 2000; Mears et al., 2001).

For cones, subtype specification is somewhat more complicated. In zebrafish, two progenitor cone populations form. One population expresses the homeobox transcription factor Six7 and can only form green cones. A second population expresses the bone morphogenetic protein (BMP) ligand Gdf6a and differentiates into three distinct subtypes of cones based on additional expression of downstream genes (Ogawa et al., 2015). If Gdf6a is solely expressed, the cells become blue cones; if Gdf6a and thyroid hormone receptor beta (Thrb) are expressed the population becomes red cones. Finally, if Gdf6a and T-box transcription factor 2b (Tbx2b) are co-expressed the population differentiates into UV cones (DuVal and Allison, 2018). In mice, which only have short- and medium-wavelength cones, disruption of Thrb results in mice deficient for M-opsin and an overabundance of S-opsin (Ng et al., 2001; Roberts et al., 2006).

Several extrinsic signaling pathways have also been shown to have a role in photoreceptor development and specific opsin expression. These factors include (but are not limited to) Fgfs, Wnts, Shh, retinoic acid (RA), Notch and thyroid hormone (T3) and changes in their morphogen concentration gradients lead to loss of photoreceptors and differential opsin expression patterns (Roberts et al., 2006; Stevens et al., 2011). Briefly, in zebrafish, Hedgehog signaling from the RPE is associated with the wave-like differentiation of photoreceptors and when disrupted, differentiation is halted resulting in a decrease in photoreceptors (Stenkamp et al., 2000). In chick and rat *in vitro* experiments, retinoic acid was shown to be essential for differentiation and survival of photoreceptors and recently has been shown to accelerate photoreceptor development in retinal organoids (Stenkamp et al., 1993; Kelley et al., 1999; Zerti et al., 2020). Furthermore, retinoic acid-treated zebrafish showed altered photoreceptor differentiation, resulting in an increased number of rod photoreceptors and red cones, and a decrease in number of blue and UV cone photoreceptors (Hyatt et al., 1996; Prabhudesai et al., 2005). Finally, Notch signaling has been shown to maintain proliferative potential of retinal progenitors and the transition to photoreceptor precursors. An early loss of Notch signaling has been shown to lead to an increase in the number of cones and later loss of signaling leads to an increased number of rods (Jadhav et al., 2006).

In summary, while much has been learned about the intrinsic transcription factors and the external signaling that influences photoreceptor development, gaps in our knowledge about the contribution and timing of these genes and factors, as well as additional photoreceptor genes that participate in this process, still remain to be addressed.

## 5 Congenital ocular defects and syndromic disorders

Development of ocular structures and retinal cells require precise spatial and temporal organization. When these spatial or temporal dynamics are disrupted, congenital ocular and retinal defects can lead to visual impairment. These defects include three main groups: optic cup, anterior segment, and retinal disorders (Table 1). Optic cup abnormalities are present when formation of the eye field, and/or proper bilateralization and evagination are disrupted leading to anophthalmia (absence of the eye), or microphthalmia (smaller than normal eye) (Mathers et al., 1997; Fantes et al., 2003). Coloboma is also a part of this group and is a consequence of the optic fissure not closing properly, which affects the iris, choroid, retina, and optic nerve (Skalicky et al., 2013). Anterior segment deficits involve the structures of the cornea, iris, ciliary body, and lens leading to aniridia, anterior segment dysgenesis, Axenfeld-Rieger anomaly, and Peters Anomaly (Smith, 2000; Semina, 2001; Komatireddy et al., 2003). Retinal disorders are those that effect the neurons of the retina and most commonly the photoreceptors. These defects include cone-rod dystrophy, Leber congenital amaurosis, and retinitis pigmentosa and result when the genes responsible for function and maintenance of the photoreceptors are disrupted leading to photoreceptor degeneration, or when the photoreceptors don’t form normally during development (Morimura et al., 1998; Sohocki et al., 1998; Lotery, 2001; Fazzi et al., 2003).

**TABLE 1:** Congenital ocular defects.

	Major characteristics	Additional ocular characteristics	Major genes	Frequency
Anterior segment defects				
Aniridia	Loss of Iris	Misshapen pupil, glaucoma, cataracts	*PAX6, FOXC1, CYP1B1*	1 in 50,000 to 100,000
Anterior segment dysgenesis	Underdeveloped iris, cornea defects, ciliary body defects, lens defects	Glaucoma, cataracts	More than 15 genes; *PITX2, FOXC1, PITX3*	Varies
Axenfeld-Rieger anomaly	Defects in iris and pupil	Glaucoma, cataracts	*PITX2* and *FOXC1*	1 in 200,000
Peters anomaly	Opaque cornea	Amblyopia, glaucoma, cataracts	*FOXC1, PAX6, PITX2, CYP1B1*	3 to 6 in 100,000
Optic cup defects				
Anophthalmia	Loss of one or both eyes	n/a	More than 75 genes; *SOX2, RAX, OTX2, PAX6*	1 in 20,000
Coloboma	Lack of optic fissure closure	Cataracts, glaucoma, myopia, nystagmus, retinal detachments	More than 50 genes; *SHH, PAX6, GDF3, VAX2*	1 in 10,000
Microphthalmia	Small eye	Cataracts, microcornea	More than 50 genes; *RAX, SIX6, OTX2, SHH, SOX2*	1 in 10,000
Retinal cell defects				
Cone—rod dystrophy	Loss of cones then rod photoreceptors	Nystagmus	More than 30 genes; *ABCA4*	1 in 30,000 to 40,000
Leber congenital amaurosis	Loss of photoreceptors	Nystagmus, keratoconus, poor pupillary reflex	More than 15 genes; *CEP290, CRB1, RPE65*	2 to 3 in 100,000
Retinitis pigmentosa	Loss of rods then cone photoreceptors	n/a	More than 60 genes; *RHO, USH2A*	1in 3,000 to 4,000
Usher syndrome	Loss of rods then cone photoreceptors	n/a	*MYO7A, CDH23,CLRN, USH2A*	4 to 17 in 100,000 people
Bardet-Biedl syndrome	Loss of cones then rod photoreceptors	n/a	More than 15 genes; BBS1, BBS10,	1 in 140,000 to 1 in 160,000
Joubert syndrome	Loss of rods then cone photoreceptors	Coloboma	More than 30 genes; *CEP290, KIF7, NPHP1*	1 in 80,000 to 1 in 100,000
Senior-Løken syndrome	Loss of photoreceptors	Nystagmus, keratoconus, poor pupillary reflex	*CEP290, NPHP1,WDR19,NPHP4*	1 in 1,000,000

While some of these defects are rare, a child’s vision and their future development can be strongly impacted. In fact, coloboma accounts for up to 10% of pediatric blindness (Chang et al., 2006). In addition, these congenital defects may present as the sole developmental defect or as part of a larger syndromic disorder. Axenfeld-Rieger anomaly and Peters Anomaly can be part of larger syndromes (Axenfeld-Rieger syndrome and Peters plus syndrome), in which affected individuals have both eye anterior segment defects in addition to craniofacial defects, dental anomalies, short stature, and intellectual disability (de Almeida and Llerena, 1993; Chang et al., 2012). Retinal cell defects are present in syndromic disorders such as Usher syndrome, Bardet-Biedl, Joubert, and Senior-Løken syndrome. In Usher syndrome, the affected individual has retinitis pigmentosa and faces a range of hearing loss and balance concerns that vary in severity depending on the affected gene (Bonnet and El-Amraoui, 2012). In Bardet-Biedl syndrome, most affected individuals have cone-rod dystrophy and are legally blind by adolescence, in addition to other characteristics such as obesity, polydactyly (extra fingers and toes), intellectual disability, and genital abnormalities (Beales, 2005). Individuals with Joubert syndrome can display brain abnormalities along with muscle, skeletal, kidney, liver and retinal dystrophy (Parisi, 2009). Senior-Løken syndrome is characterized by the combination of Leber congenital amaurosis and loss of photoreceptors with the kidney condition of nephronophthisis (Ronquillo et al., 2012). Finally, microphthalmia, anophthalmia, and coloboma, collectively referred to as MAC, can occur in isolation or alongside those of brain and craniofacial defects, signifying the similar signaling pathways responsible for the formation of head structures. One example is CHARGE syndrome, a genetic neurocristopathy characterized by coloboma, heart defects, choanal atresia, growth retardation, genital abnormalities, and ear abnormalities (Hsu et al., 2014).

## 6 Clinical presentation of CHARGE syndrome

**FIGURE 3:**
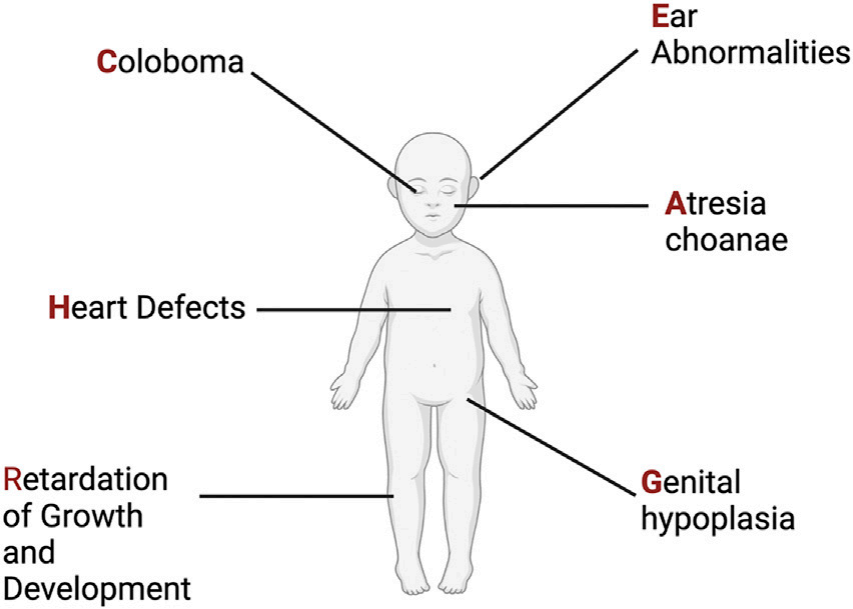
CHARGE Syndrome. Original phenotypic description leading to the acronym for CHARGE Syndrome. See text for more details. Figure created with Biorender.com.

The syndrome now known as CHARGE was first described in 1979 through two different cohorts of individuals with ear, ocular, cardiac, craniofacial anomalies, and intellectual disability (Hall, 1979; Hittner et al., 1979; Issekutz et al., 2005; Janssen et al., 2012). Two years later another cohort of individuals was described, and the publishing group proposed the acronym CHARGE: C-coloboma, H-heart disorders A-atresia choanae, R-retarded growth and retarded development and/or CNS anomalies, G-genital hypoplasia, and E-ear anomalies and/or deafness (Pagon et al., 1981) (Figure 3). The prevalence of CHARGE syndrome is estimated to be between 1 in 10,000 to 1 in 15,000 live births.

Since first described, phenotypic reports have expanded, resulting in more detail and a wider range of possible phenotypic presentations. Some of the clinical manifestations of CHARGE syndrome are present at birth, whereas others become apparent later in life or upon more in-depth examination and testing. Ocular defects include coloboma and microphthalmia and will be discussed in more detail in Section 8 (Nishina et al., 2012). Heart defects include a wide spectrum of congenital abnormalities including aortic arch interruption, tetralogy of Fallot, double outlet right ventricle, arch vessel anomalies, and atrioventricular septal defects (Corsten-Janssen et al., 2013). Craniofacial and upper respiratory malformations include choanal atresia (narrowing of nasal cavity), orofacial clefts, tracheoesophageal fistulas along with characteristic facial features of prominent foreheads and nasal bridges (Inchingolo et al., 2014). Growth and developmental anomalies involve central nervous defects, cranial nerve abnormalities, short stature, and intellectual delays (Graham et al., 2005; Blake et al., 2008; Dörr et al., 2015; Hoch et al., 2017). Genital abnormalities include genital hypoplasia and late puberty (Ragan et al., 1999). Ear abnormalities involve cup shaped outer ear, triangular concha, ossicular malformations, absence or hypoplasia of semicircular canals and sensory hearing deficits (Morimoto et al., 2006; Holcomb et al., 2013; Ha et al., 2016; Wineland et al., 2017).

The expansion of the phenotypes described can be partially attributed to the identification in 2004 of the major causative gene, *CHD7*, which is reported to be mutant in 70%–90% of CHARGE syndrome cases (Vissers et al., 2004; Jongmans et al., 2006; Zentner et al., 2010). While molecular diagnosis was initially performed via traditional Sanger sequencing of *CHD7*, the recent application of whole exome sequencing in individuals suspected of having CHARGE syndrome has provided an opportunity to expand the number of pathogenic variants that have been detected in *CHD7*, as well as identifying additional genes associated with this disorder. The wide spectrum of phenotypes that have now been described for individuals with *CHD7*-associated CHARGE syndrome has resulted in several different published clinical diagnostic criteria centered on major and minor characteristics (Blake et al., 1998; Verloes, 2005; Blake and Prasad, 2006). The latest proposed criteria suggest a CHARGE diagnosis based on 2 major and any number of minor characteristics (Hale et al., 2016) (Table 2). One of the major characteristics is a pathogenic variant in *CHD7*, which has been the focus of CHARGE Syndrome research to further understand the diverse spectrum and mechanism of phenotypes observed and will be discussed more in Section 7.

**TABLE 2:** CHARGE syndrome diagnostic criteria.

CHARGE syndrome diagnostic criteria	
Major (2 or More)	Minor
Coloboma	Cranial nerve dysfunction
Choanal atresia or cleft palate	Dysphagia/feeding difficulties
Abnormal external, middle, or inner ears	Structural brain anomalies
Pathogenic *CHD7* variant	Developmental delay
	Hypothalamo-hypophyseal dysfunction (gonadotropin or growth hormone deficiency) and genital anomalies
	Heart or esophagus malformation
	Renal anomalies
	Skeletal and limb anomalies

## 7 Chromodomain helicase DNA binding protein 7 and CHARGE syndrome

CHD7 is a member of the chromodomain helicase-DNA binding domain (CHD) family of proteins. CHD members have specific non-redundant roles in the processes involved in chromatin manipulation using ATP-hydrolysis (Woodage et al., 1997; Flaus and Owen-Hughes, 2011; Manning and Yusufzai, 2017). Members of this family have two conserved motifs: two chromodomains located in the N-terminal region, and the SNF2-like ATPase domains located in the central region, with other domains less well characterized at the C-terminus (Delmas et al., 1993). For a more in-depth discussion of CHD7 functional domains, we refer the reader to previously published reviews (Marfella and Imbalzano, 2007; Layman et al., 2010). In general, CHD7 has been shown to be responsible for nucleosome mobility, recognizing unique target sites to allow for the removal and sliding of the histone octamer thus leading to DNA access (Bouazoune and Kingston, 2012). This chromatin manipulation is essential for DNA transcription, replication, and repair, making CHD7 a vital gene for normal development (Ho and Crabtree, 2010). CHD7 has been shown to preferentially associate with H3Kme1, H3K4me3, and H3K27ac histone modifications, which mark poised and active promoters and enhancers (Reddy et al., 2021). ChIP experiments have shown that CHD7 occupies distinct sets of binding sites in different cell types, indicating that CHD7 regulates chromatin accessibility in a tissue-specific manner (Schnetz et al., 2009). CHD7 may control target gene expression by acting solely or as member of a complex with other transcription factors, such as PBAF and CHD8, which was shown to interact wth CHD7 via its conserved SANT domain (Bajpai et al., 2010; Batsukh et al., 2010). CHD7-mediated tissue-specific gene expression control has been found to be essential for cellular proliferation and differentiation in addition to maintenance of adult neural stem cell populations (Martin, 2010; Jones et al., 2015).

Heterozygous pathogenic variants in *CHD7* are identified in 70%–90% of clinically diagnosed individuals with CHARGE (Vissers et al., 2004; Jongmans et al., 2006; Zentner et al., 2010). With over 500 pathogenic variants spanning the entirety of the 38-exon gene (2997 amino acids, ∼336 kD protein), 90% of the variants are nonsense, frameshift, and splice site variants resulting in a truncated protein (Bergman et al., 2011). In addition, pathogenic variants in *CHD7* have been reported in clinical cases of Kallmann syndrome, which is a disorder involving hypogonadotropic hypogonadism (deficit in gonadotropin-releasing hormone) and hyposmia or anosmia (diminished or lack of smell) (Jongmans et al., 2009). Interestingly, 70% of variants found in *CHD7* causing Kallmann syndrome are missense variants (Marcos et al., 2014). Since identification of *CHD7* as a causative gene in CHARGE syndrome, research has focused on understanding its role in the affected tissues. The following sections will focus on the ocular complications of CHARGE syndrome and the role that CHD7 plays in the development of the eye.

## 8 Ocular complications of CHARGE syndrome

Clinical reports indicate that coloboma is present in over 80% of clinically diagnosed individuals with CHARGE. The colobomas range in severity and involvement of the retina, choroid, lens, and iris. While the majority of the observed colobomas are bilateral, some individuals present with unilateral ocular defects. In addition to coloboma, other ocular complications including microphthalmia, optic nerve hypoplasia, nystagmus, cataracts, amblyopia, microcornea, strabismus and rarely angle closure defects and retinal detachment have been reported (Russell-Eggitt et al., 1990; Tellier et al., 1998; Guirgis and Lueder, 2003; Strömland et al., 2005; Aramaki et al., 2006; Lalani et al., 2006; Natung et al., 2014; Martin et al., 2020).

While ocular structural defects have been identified in different case reports and phenotype reviews of CHARGE syndrome, we lack an understanding of the correlation between the extent of ocular defects and an individual’s visual impairment. This is partially due to the complex clinical presentation of CHARGE syndrome, which often imposes limits on performing normal vision assessment methods. Recently several groups have tried to address this lack of connection. First, the VISIOCHARGE questionnaire combined ophthalmological findings with a self- and/or guardian-administered questionnaire assessing distance-vision, near-vision, and overall vision. This group concluded that visual skills of everyday life were relatively good even in the presence of severe ocular structural defects, such as bilateral colobomas, and there was no association between visual ability and the severity of ocular malformation (Martin et al., 2020). However, this study has its limitations based on the small size of the cohort and the nature of self-administered questionnaires. More recently, the VISIOCHARGE questionnaire was combined with adapted visual behavior assessments and ophthalmic assessments to better fit the complexity of clinical findings in CHARGE syndrome (Onesimo et al., 2021). Visual behavior assessments were adapted to measure the ability to fix, track, perform saccades, and assess visual acuity and visual fields along with measuring contrast sensitivity, stereopsis, and strabismus. Overall, this systematic assessment resulted in categorizing 57% of the individuals with severe visual impairment. However, there was no correlation between the extent of visual impairment and the severity of the ocular structural abnormality. This study did identify a correlation between ocular abnormalities and the type of pathogenic variants in CHD7, suggesting that earlier truncating pathogenic variants resulted in more severe findings such as extensive colobomas. This study also has limitations due to size, but leads to questions about why there is a lack of correlation between ocular structural defects and assessed visual function. One possible explanation is that loss of CHD7 results in additional retinal abnormalities that are currently underdiagnosed in CHARGE syndrome. To further understand the full extent of ocular complications of CHARGE syndrome, it is essential to better understand CHD7’s role in eye development beyond the initial stage of ocular morphogenesis.

## 9 Role of CHD7 in development of ocular structures

Xenopus, Drosophila, zebrafish, and mouse models have been established to further study the development and mechanism of CHD7 and CHARGE syndrome (Bajpai et al., 2010; Melicharek et al., 2010; Patten et al., 2012). Homozygous loss-of-function mouse models are embryonic lethal at E10.5 while heterozygous mice display characteristics like those of individuals with CHARGE (Bosman et al., 2005; Hurd et al., 2007). These models have been used to extensively study brain, ear, heart, and craniofacial developmental phenotypes upon loss of CHD7 (Bosman et al., 2005; Melicharek et al., 2010; Ogier et al., 2014; Sperry et al., 2014; Whittaker et al., 2017a). However, less work has concentrated on eye development in these models, partially due to the complex contributions of multiple tissues in the eye.

**FIGURE 4:**
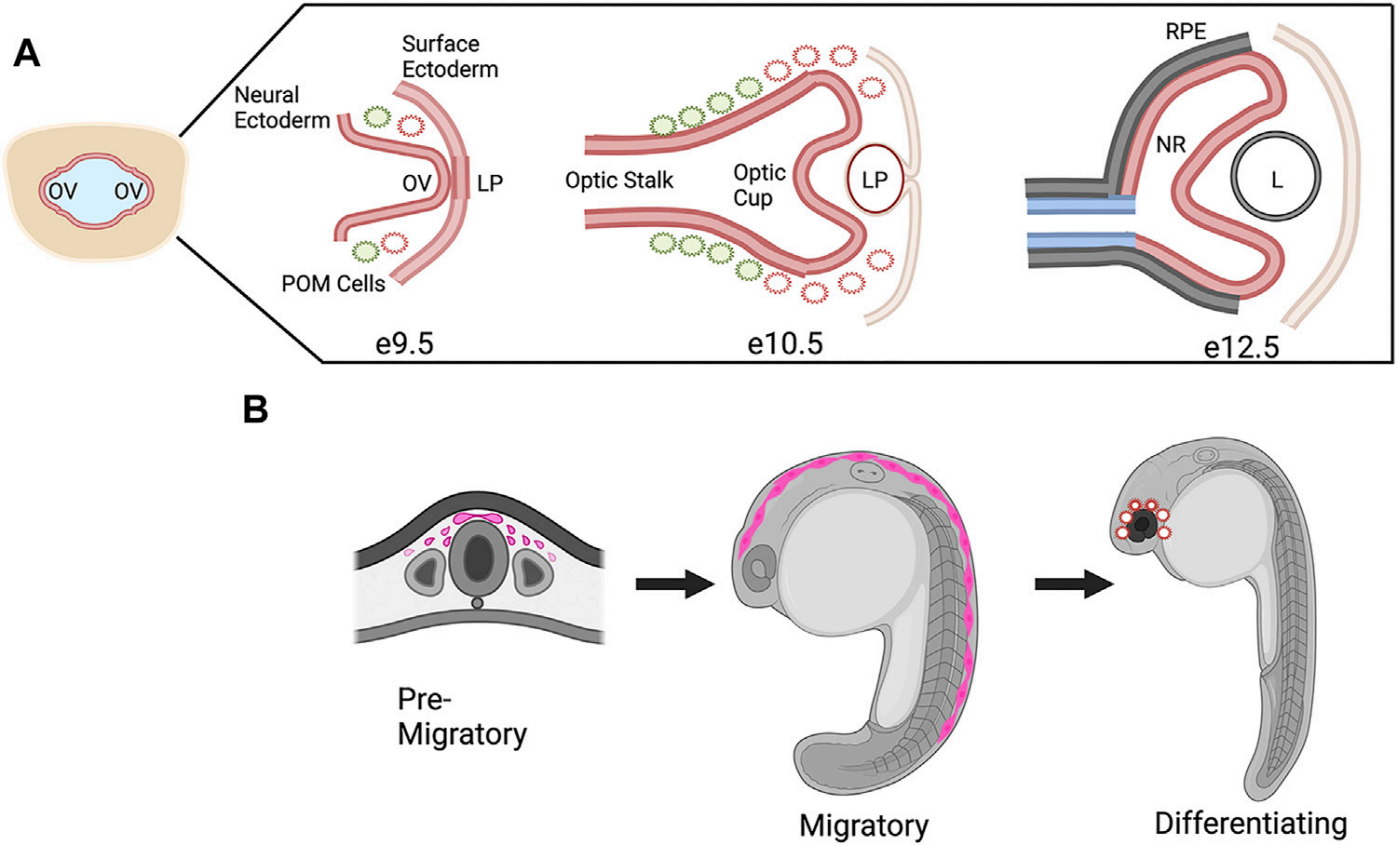
CHD7 Expression in Ocular Morphogenesis and Neural Crest Cell Development. **(A)** Schematic representation of ocular development stages in mouse from optic vesicle evagination at E9.9 to bi-layered optic cup at E12.5, CHD7 expression is colored in red. OV, optic vesicle; LP, lens placode/pit; POM, periocular mesenchyme; RPE, retinal pigment epithelium; NR, neural retina; L, lens. **(B)** Schematic representation of CHD7 expression in neural crest cell development, CHD7 expression is colored in pink. Figure created with Biorender.com.

One study that focused on CHD7 in mouse eye development used conditional knockouts to delete *Chd7* from various embryonic tissues that contribute to the eye, including the neural ectoderm (*Rx-Cre*), surface ectoderm (*Le-Cre*), and both surface and neural ectoderm (*Foxg1-Cre*) (Gage et al., 2015). This work was the first detailed expression study in the eye and CHD7 was shown to be widely expressed in early neural and surface ectoderm from E9.5-E12.5, the timing of which corresponds to optic vesicle evagination at E9.5 through formation of the optic cup and lens at E12.5 (Figure 4A). Furthermore, conditional knockouts of *Chd7* in surface ectoderm and/or neural ectoderm demonstrated that expression of CHD7 was required in the neural ectoderm for proper ocular morphogenesis of the optic cup and optic fissure closure. In addition, this work established and qualitatively described the presence of coloboma in the *Chd7* mutant mouse model which previously had not been addressed.

Ocular structural defects upon loss of Chd7 in other species have been broadly described but not rigorously investigated. Results of knockdown and knockout studies contain general descriptions of microphthalmia, coloboma, and anterior defects, but an in-depth understanding of mechanism or contribution of CHD7 to these defects is lacking (Jacobs-Mcdaniels and Albertson, 2011; Balow et al., 2013; Cloney et al., 2018; Liu et al., 2018; Liu and Liu, 2019; Breuer et al., 2020).

An area of focus for CHD7 research has been its role in neural crest cells, which contribute to many of the structures affected in CHARGE syndrome (Siebert et al., 1985). Neural crest cells are multipotent migratory cells that arise early in development and contribute to tissues and structures in the heart, craniofacial skeleton, ear, eye, and peripheral nervous system, among others (Pauli et al., 2017). It has been shown in mouse, zebrafish, and Xenopus models that Chd7 is expressed in pre-migratory neural crest cells and when Chd7 expression is altered there is a decrease and disruption of these cells (Fujita et al., 2014; Schulz et al., 2014). Expression continues during neural crest cell migration where in Xenopus and in human cells (including patient derived iPS cells) CHD7 has been shown to target downstream genes such as *Sox9*, *Twist*, and *Snail 1/2* (Bajpai et al., 2010; Sperry et al., 2014; Okuno et al., 2017). Furthermore, in mouse models CHD7 has been shown to work in conjunction with other transcription factors and chromatin remodeling complexes to direct neural crest cell differentiation and when CHD7 is absent neural crest cell fates are altered (Figure 4B) (Fan et al., 2021).

Concentrating on the eye, it is known that cranial neural crest cells contribute to the periocular mesenchyme, which forms anterior ocular structures including the cornea, ciliary body, iris, sclera, and aqueous humor outflow tract. Disruptions in neural crest cells result in defects of these structures and congenital disorders such as Axenfeld-Reiger syndrome and Peters Anomaly (Cvekl and Tamm, 2004; Gage et al., 2005). Neural crest cells also secondarily play a role in optic cup formation and optic fissure closure, interacting with the neural derived optic cup. Disruptions in this process result in microphthalmia and coloboma (Bassett et al., 2010; Bryan et al., 2020).

However, the exact role of CHD7 in the cranial neural crest cells that contribute to ocular development is still unknown, creating a need for more investigation and additional models. Anterior segment defects have been identified in individuals with CHARGE suggesting that CHD7 may contribute to neural crest cells in the periocular mesenchyme (McMain et al., 2008; Nishina et al., 2012; Dote et al., 2019). Models with live imaging and transgenic lines of zebrafish that have fluorescently labeled neural crest cells could help to delineate the exact role of Chd7 in cranial neural crest cells and periocular mesenchyme. Furthermore, based on CHD7’s role in neural ectoderm and ocular structures, conditional knockout models will need to be established to determine the contribution from each cell type.

## 10 CHD7 in neurogenesis

In addition to the role of CHD7 in neural crest cell dynamics, CHD7 has strongly been associated with tissue-specific neurogenesis. These associations are evident in many of the systems affected in CHARGE syndrome which contain nervous tissue (Table 3). In the ear, CHD7 has been shown to act upstream of pro-neural genes in inner ear neuroblasts; mice lacking functional CHD7 have a smaller vestibulo-cochlear ganglion and decrease in neuron number (Hurd et al., 2010). In the olfactory system, CHD7 is expressed in the pro-neural basal cells during development and adulthood. When disrupted, there is disorganization and a decrease in olfactory sensory neurons (Layman et al., 2009). Gonadotropin-releasing hormone (GnRH) neurons are also reduced in the hypothalamus of haploinsufficient CHD7 mice (Layman et al., 2011). In other parts of the brain, there are examples of CHD7 controlling neurogenesis in both development and adulthood. The neurogenic areas of adult mammalian brain, the subventricular zone (SVZ) of the lateral ventricle and the subgranular zone (SGZ) of the dentate gyrus in the hippocampus, show expression of CHD7 in active neural stem cells and when inactivated there is a decrease in adult neurogenesis (Feng et al., 2013). During development, CHD7 is expressed in cerebellar granule cells and loss of CHD7 in subsets of these cells leads to a decrease of neurogenesis and eventual cerebellar hypoplasia (Whittaker et al., 2017b, 2017a; Donovan et al., 2017; Feng et al., 2017).

**TABLE 3:** CHD7 and neurogenesis.

System	Neurons affected	Potential downstream targets of CHD7	Association with CHARGE phenotype
Cerebellum	Reduction in cerebellar granule neurons	*Reln, Fgf8*	Cerebellar hypoplasia leading to developmental delays
Global brain	Reduction in GABAergic neurons	*paqr3b*	Autism-like behavior, attention-deficit/hyperactivity disorder, anxiety, aggressivity and seizures
Hippocampus	Decrease in adult neurogenesis from subventricular zone (SVZ) of the lateral ventricle and the subgranular zone (SGZ) of the dentate gyrus	*Sox4, Sox11*	Development delays
Hypothalamus	Reduced gonadotropin-releasing hormone (GnRH) neurons	*Fgfr1,Bmp4, Otx2*	Hypogonads, genital hypoplasia, and delayed puberty
Olfactory	Reduced olfactory bulb size and reduced olfactory neurons	*Mash1, NeuroD*	Hyposmia and Anosmia
Auditory and vestibular	Vestibulo-cochlear ganglion size and neuron number	*Ngn1, Otx2, Fgf10*	Hearing loss and balance disorders

Globally, CHD7 has been found to be a co-factor of Sox2, which is essential for neural stem cell maintenance and neurogenesis in mouse. It also contributes to neural progenitor differentiation in mouse embryonic stem cells where the loss of CHD7 results in disruption of the number and complexity of new neurons (Engelen et al., 2011; Yao et al., 2020). In addition to mouse models, recent *chd7* mutant and morphant zebrafish models have shown a decrease in branchiomotor neurons of the hindbrain and GABAergic neurons in the brain (Patten et al., 2012; Jamadagni et al., 2021).

While the effects of *CHD7* pathogenic variants on neurogenesis have been studied for many regions of the central and peripheral nervous system, there is little data available on whether loss of CHD7 alters retinal neurogenesis. As described above, much of the focus of published literature has been on ocular morphogenesis and those studies did not examine later timepoints that are relevant for retinal neuron differentiation (Gage et al., 2015).

**FIGURE 5:**
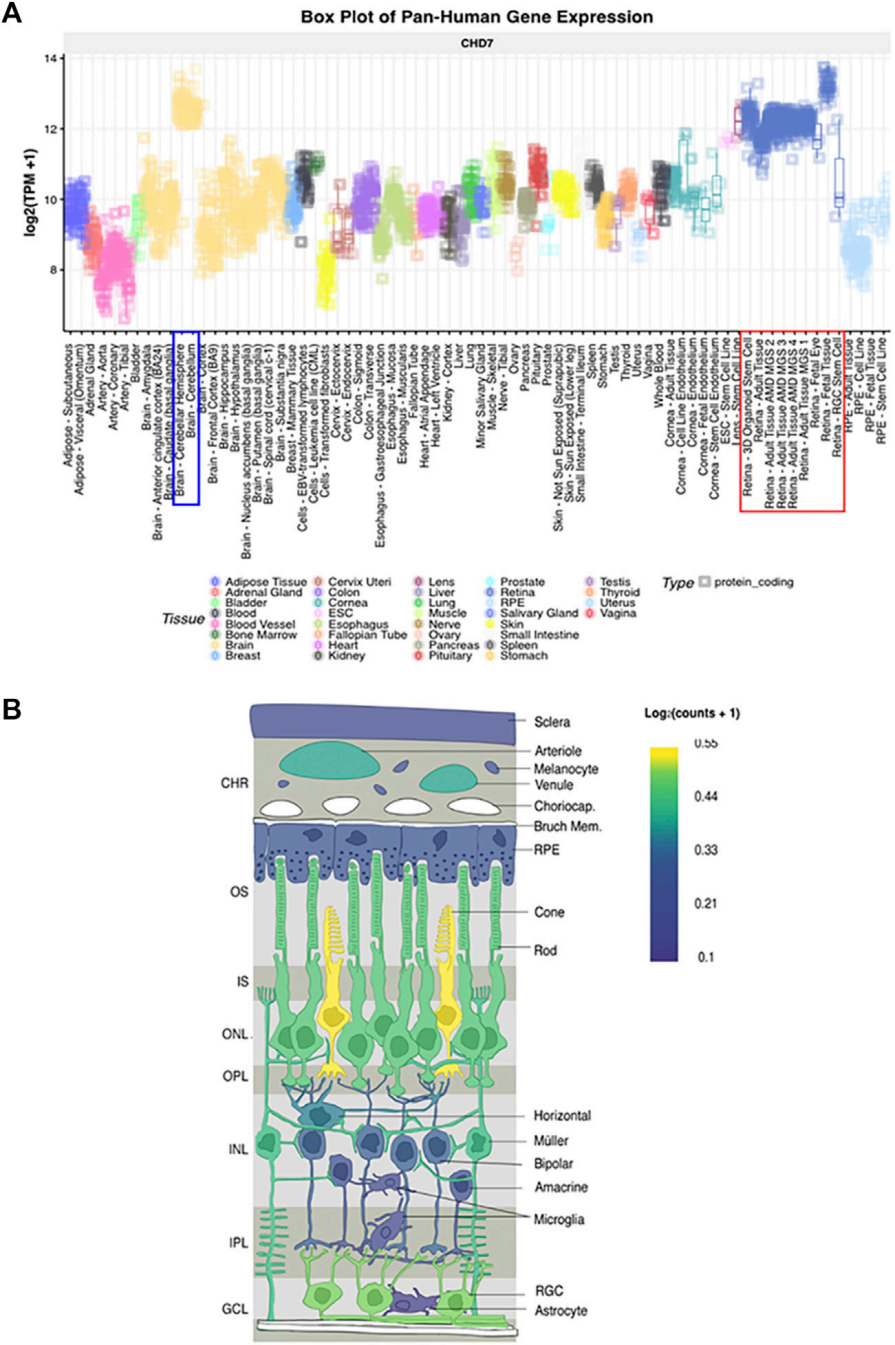
CHD7 expression in human retina **(A)** Box plot of pan-Human *CHD7* expression in fetal and adult retinal tissue (red outline) compared to other tissues, assembled with the eyeIntegration v1.05 platform (Bryan et al., 2018). **(B)** *In-situ* projection from published human single cell RNAseq datasets. Assembled with PLatform for Analysis of scEiad (Swamy et al., 2021). Expression is highest in cones, rods, and retinal ganglion cells with lower expression in Müller glia. Reprinted with permission from (Krueger et al., 2022).

With the advancement of single-cell transcriptomics, large data meta-analysis platforms have become more accessible to analyze specific tissue transcriptomics. This is the case with the eye and the analysis platform eyeIntegration v1.05 from the National Eye Institute which collates human eye tissue RNA-seq data with other human body tissues (Bryan et al., 2018). When using this platform, Chd7 is shown to be globally expressed in fetal human retina and postnatal retina at levels equal to that of the cerebellum (Figure 5 and Krueger et al., 2022). These data suggest that CHD7 may have some functional role in both the developing and mature retina. Additional data supporting a role for CHD7 in the retina comes from GWAS studies, which have identified variants in *CHD7* that are significantly associated with myopia (Kiefer et al., 2013; Verhoeven et al., 2013; Tedja et al., 2018). Given that retinal signals drive eye growth (Brown et al., 2022), and abnormalities in the photoreceptor cell layer are associated with myopia development (Greenwald et al., 2017; Chakraborty et al., 2019), this suggests that altered CHD7 expression or function may contribute to a broader spectrum of retinal defects than has been previously appreciated.

Interestingly, morpholino-mediated knockdown of zebrafish *chd7* was associated with a decrease in retinal ganglion cells and photoreceptors in addition to global retinal disorganization and laminar defects (Patten et al., 2012). However, there have been no follow up studies to further characterize this phenotype or elucidate contributions of Chd7 to the retina. Taken together with the lack of correlation between the presence of ocular malformations and extent of visual impairment in individuals with CHARGE, this may point to retinal neuron deficits as an additional feature of CHARGE syndrome, and thus should be an avenue for future studies. Furthermore, recent work has shown that Chd7 plays a role in protecting hair cells of the ear from oxidative stress and loss of Chd7 leads to misregulation of stress response pathways resulting in degeneration (Ahmed et al., 2021). Given that photoreceptors are sensory neurons with a high metabolic demand, the role of Chd7 in their oxidative stress response also warrants investigation.

## 11 SoxC factors: Potential targets of CHD7 in eye development

The wide spectrum of phenotypes observed in CHARGE syndrome is not only a result of the broad expression of CHD7 in development but also the wide range of potential downstream targets of CHD7 transcriptional regulation. Generally, CHD7 has been shown to bind regions distal to transcription start sites in the genome marked by H3K4 methylation—the histone modification indicating active transcription. This epigenetic signature of histone methylation and correlation with CHD7 binding signifies the role CHD7 plays in modifying downstream gene expression (Schnetz et al., 2009). Two of these identified binding sites are in the promoters of *Sox11* and *Sox4*, which appears to be required during adult neurogenesis in the hippocampus. In CHD7 mutant mice, there is a decrease in the active histone marker H3K4 methylation at the *Sox11* and *Sox4* promoters in neural stem cells potentially as a direct result of the coordination of Chd7 with methyltransferase complexes (Yan et al., 2020) . *Sox4* and *Sox11* were also shown to be direct targets of CHD7 binding via chromatin immunoprecipitation (ChIP) assays in cultured neural stem cells. In *CHD7* mutants the chromatin structure at *Sox11* and *Sox4* loci remains closed, preventing the transcription of *Sox11* and *Sox4* and inhibiting neurogenesis (Feng et al., 2013).

SOX4 and SOX11 are members of the group C family of SOX transcriptional activators, whose name is derived from a shared DNA binding domain (the SRY-box) originally identified in the mammalian sex-determining gene SRY (Denny et al., 1992). SOX proteins encode a large family of transcription factors that are grouped into individual families, SOXA-SOXH, based on protein structure, expression, and conservation of amino acids. Within individual families there is homology among the N-terminal DNA binding high-mobility group (HMG) region, in addition to regions outside of this domain. The family of SOXC proteins also contains SOX12. All three genes are single exon genes that encode two highly conserved domains, the HMG domain and a C-terminal transactivation domain (TAD) (Bowles et al., 2000). The HMG domain enables specific DNA binding to the sequence (A/T A/T CAA A/T) as well as nonspecific binding in the minor groove of DNA, which induces DNA bending and makes regulatory regions such as promoters and enhancers more accessible to other proteins (Harley et al., 1994; Wegner, 1999; Kamachi et al., 2000). These domains allow for transcriptional control of downstream targets with the contribution of other regulatory proteins.

SOX proteins have been shown to regulate many developmental processes, including early control of the establishment of blastocyst, gastrulation, and germ layers as well as continued cellular pluripotency and cell fate and differentiation in tissue formation and organogenesis (Kamachi et al., 2000; Lefebvre et al., 2007; She and Yang, 2015). More specifically, SOXC proteins have been implicated in different aspects of neurogenesis including fate determination, specification, migration, tract formation, and plasticity (Bhattaram et al., 2010; Chen et al., 2015).

Interestingly, Sox4 and Sox11 are also expressed in neural crest cells during development and are strongly expressed in purified neural crest cells from zebrafish (Uy et al., 2015). Moreover, Sox4- and Sox11-deficient animal models display coloboma, cardiac malformations and brain defects like those seen in CHARGE syndrome (Wurm et al., 2008; Paul et al., 2014; Pillai-Kastoori et al., 2014; Schulz et al., 2014; Gnedeva and Hudspeth, 2015; Wen et al., 2015). In addition, our group has previously shown that Sox11 contributes to coloboma in zebrafish and humans and another group has identified an individual with CHARGE with a chromosomal duplication involving the SOX11 locus (Pillai-Kastoori et al., 2014; Sperry et al., 2016). To better understand the connection of CHD7 and Sox11 with respect to the ocular features of CHARGE syndrome, further characterization of the contribution of these specific genes to ocular development and retinal neurogenesis must be elucidated.

## 12 Conclusion

The intricate process of visual system development requires the coordination of numerous signaling pathways and gene expression programs across different embryonic tissues to achieve a fully functional ocular structure. Disruptions to this process can affect many parts of the developing eye, and several result in significant pediatric visual impairment. One example is CHARGE syndrome, a genetic disorder characterized by ocular coloboma, heart defects, choanal atresia, growth retardation, genital abnormalities, and ear abnormalities. Pathogenic variants in the chromatin remodeling factor CHD7 are the most common cause of CHARGE syndrome, accounting for approximately 70%–90% of CHARGE syndrome cases. Coloboma, which is present in over 80% of individuals with CHARGE syndrome, is generally thought to be the major ocular complication of CHARGE syndrome. However, CHARGE syndrome is also associated with other ocular complications, including microphthalmia, optic nerve hypoplasia, nystagmus, cataracts, amblyopia, microcornea, strabismus, angle closure defects, and retinal detachment. Moreover, the lack of correlation between the extent of visual impairment and the presence of structural ocular defects indicates that additional ophthalmic complications associated with CHARGE syndrome may be missed or overlooked. One defining feature of CHARGE syndrome is that it involves abnormal neural crest cell development: the structures affected in CHARGE syndrome—eyes, brain, heart, craniofacial structures, ear—are composed of neural crest cell derivatives; *CHD7* is expressed in neural crest cells; and neural crest cell progenitors are reduced and their migration is abnormal in animal models of CHARGE syndrome. Therefore, it is possible that some of the ocular defects associated with CHARGE syndrome are due to cell autonomous functions of CHD7 in retnal progenitor cells, whereas some ocular complications could result from non-cell autonomous mechanisms due to loss of CHD7 in cranial neural crest cells and/or the periocular mesenchyme, although the precise balance of mechanisms remain to be determined. Future studies could address this question using conditional knockout models and fluorescent transgenic reporter lines that label specific neural crest cell lineages. CHARGE syndrome also involves abnormal neurogenesis in the CNS (which includes the retina). Recent studies in animal models of CHD7 deficiency, as well as GWAS studies implicating CHD7 variants in myopia, point to a potential role for CHD7 in the developing retina and in retinal cell type differentiation or maintenance. Therefore, future studies should more closely investigate the function of CHD7 in specific subsets of retinal neurons, in particular the critical light-capturing photoreceptors. One promising approach to modeling the ocular complications of CHARGE syndrome would be to use patient-derived iPS cells to generate retinal organoids. A better understanding of the pathogenetic mechanisms underlying the ocular complications of CHARGE syndrome is critical, not only to improve patient care and to identify potential long-term disease sequelae, but also to inform the development of new therapeutic strategies.
